# Supplementary material for: A Critical View of the Application of the APEX Software (Aqueous Photochemistry of Environmentally-Occurring Xenobiotics) to Predict Photoreaction Kinetics in Surface Freshwaters
Source: Molecules. 2019 Dec 18;25(1):9. doi: 10.3390/molecules25010009 (PMC7017383; doi:10.3390/molecules25010009)
Supplement: Supplementary file 1 [file molecules-25-00009-s001.zip › 3.2.4_gcc-4.4.0.zip!/3.2.4_gcc-4.4.0/3.2.4_gcc-4.4.0/doc/octave/pdf/refcard-a4.pdf]

Octave Quick Reference

Octave Version 3.0.0

Starting Octave

octave	start interactive Octave session
octave *file*	run Octave on commands in *file*
octave --eval *code*	Evaluate *code* using Octave
octave --help	describe command line options

Stopping Octave

quit or exit	exit Octave
INTERRUPT	(*e.g.* C-c) terminate current command and return to top-level prompt

Getting Help

help	list all commands and built-in variables
help *command*	briefly describe *command*
doc	use Info to browse Octave manual
doc *command*	search for *command* in Octave manual
lookfor *str*	search for *command* based on *str*

Motion in Info

SPC or C-v	scroll forward one screenful
DEL or M-v	scroll backward one screenful
C-l	redraw the display

Node Selection in Info

n	select the next node
p	select the previous node
u	select the 'up' node
t	select the 'top' node
d	select the directory node
<	select the first node in the current file
>	select the last node in the current file
g	reads the name of a node and selects it
C-x k	kills the current node

Searching in Info

s	search for a string
C-s	search forward incrementally
C-r	search backward incrementally
i	search index & go to corresponding node
,	go to next match from last 'i' command

Command-Line Cursor Motion

C-b	move back one character
C-f	move forward one character
C-a	move to the start of the line
C-e	move to the end of the line
M-f	move forward a word
M-b	move backward a word
C-l	clear screen, reprinting current line at top

Inserting or Changing Text

M-TAB	insert a tab character
DEL	delete character to the left of the cursor
C-d	delete character under the cursor
C-v	add the next character verbatim
C-t	transpose characters at the point
M-t	transpose words at the point

[ ] surround optional arguments … show one or more arguments

Killing and Yanking

C-k	kill to the end of the line
C-y	yank the most recently killed text
M-d	kill to the end of the current word
M-DEL	kill the word behind the cursor
M-y	rotate the kill ring and yank the new top

Command Completion and History

TAB	complete a command or variable name
M-?	list possible completions
RET	enter the current line
C-p	move 'up' through the history list
C-n	move 'down' through the history list
M-<	move to the first line in the history
M->	move to the last line in the history
C-r	search backward in the history list
C-s	search forward in the history list
history [-q] [*N*]	list *N* previous history lines, omitting history numbers if -q
history -w [*file*]	write history to *file* (~/.octave_hist if no *file* argument)
history -r [*file*]	read history from *file* (~/.octave_hist if no *file* argument)
edit_history *lines*	edit and then run previous commands from the history list
run_history *lines*	run previous commands from the history list
[*beg*] [*end*]	Specify the first and last history commands to edit or run.

If *beg* is greater than *end*, reverse the list of commands before editing. If *end* is omitted, select commands from *beg* to the end of the history list. If both arguments are omitted, edit the previous item in the history list.

Shell Commands

cd *dir*	change working directory to *dir*
pwd	print working directory
ls [*options*]	print directory listing
getenv (*string*)	return value of named environment variable
system (*cmd*)	execute arbitrary shell command string

Matrices

Square brackets delimit literal matrices. Commas separate elements on the same row. Semicolons separate rows. Commas may be replaced by spaces, and semicolons may be replaced by one or more newlines. Elements of a matrix may be arbitrary expressions, assuming all the dimensions agree.

[*x*, *y*, ...]	enter a row vector
[*x*; *y*; ...]	enter a column vector
[*w*, *x*; *y*, *z*]	enter a 2×2 matrix

Multi-dimensional Arrays

Multi-dimensional arrays may be created with the *cat* or *reshape* commands from two-dimensional sub-matrices.

squeeze (*arr*)	remove singleton dimensions of the array.
ndims (*arr*)	number of dimensions in the array.
permute (*arr*, *p*)	permute the dimensions of an array.
ipermute (*arr*, *p*)	array inverse permutation.
shiftdim (*arr*, *s*)	rotate the array dimensions.
circshift (*arr*, *s*)	rotate the array elements.

Sparse Matrices

sparse (...)	create a sparse matrix.
speye (*n*)	create sparse identify matrix.
sprand (*n*, *m*, *d*)	sparse rand matrix of density *d*.
spdiags (...)	sparse generalization of *diag*.
nnz (*s*)	No. non-zero elements in sparse matrix.

Ranges

base : *limit*
base : *incr* : *limit*

Specify a range of values beginning with *base* with no elements greater than *limit*. If it is omitted, the default value of *incr* is 1. Negative increments are permitted.

Strings and Common Escape Sequences

A *string constant* consists of a sequence of characters enclosed in either double-quote or single-quote marks. Strings in double-quotes allow the use of the escape sequences below.

\\	a literal backslash
\"	a literal double-quote character
\'	a literal single-quote character
\n	newline, ASCII code 10
\t	horizontal tab, ASCII code 9

Index Expressions

var (*idx*)	select elements of a vector
var (*idx1*, *idx2*)	select elements of a matrix
scalar	select row (column) corresponding to *scalar*
vector	select rows (columns) corresponding to the elements of *vector*
range	select rows (columns) corresponding to the elements of *range*
:	select all rows (columns)

Global and Persistent Variables

global *var1* ...	Declare variables global.
global *var1* = *val*	Declare variable global. Set intial value.
persistent *var1*	Declare a variable as static to a function.
persistent *var1* = *val*	Declare a variable as static to a function and set its initial value.

Global variables may be accessed inside the body of a function without having to be passed in the function parameter list provided they are declared global when used.

Selected Built-in Functions

EDITOR	editor to use with edit_history
Inf, NaN	IEEE infinity, NaN
NA	Missing value
PAGER	program to use to paginate output
ans	last result not explicitly assigned
eps	machine precision
pi	π
1i	$\sqrt{-1}$
realmax	maximum representable value
realmin	minimum representable value

Copyright 1996, 1997, 2007 John W. Eaton Permissions on back

Assignment Expressions

var = *expr*	assign expression to variable
var (*idx*) = *expr*	assign expression to indexed variable
var (*idx*) = []	delete the indexed elements.
var {*idx*} = *expr*	assign elements of a cell array.

Arithmetic and Increment Operators

x + *y*	addition
x - *y*	subtraction
x * *y*	matrix multiplication
x .* *y*	element by element multiplication
x / *y*	right division, conceptually equivalent to `(inverse (y') * x')'`
x ./ *y*	element by element right division
x \ *y*	left division, conceptually equivalent to `inverse (x) * y`
x .\ *y*	element by element left division
x ^ *y*	power operator
x .^ *y*	element by element power operator
- *x*	negation
+ *x*	unary plus (a no-op)
x '	complex conjugate transpose
x .'	transpose
++ *x* (-- *x*)	increment (decrement), return *new* value
x ++ (*x* --)	increment (decrement), return *old* value

Comparison and Boolean Operators

These operators work on an element-by-element basis. Both arguments are always evaluated.

- *x* < *y* — true if *x* is less than *y*
- *x* <= *y* — true if *x* is less than or equal to *y*
- *x* == *y* — true if *x* is equal to *y*
- *x* >= *y* — true if *x* is greater than or equal to *y*
- *x* > *y* — true if *x* is greater than *y*
- *x* != *y* — true if *x* is not equal to *y*
- *x* & *y* — true if both *x* and *y* are true
- *x* | *y* — true if at least one of *x* or *y* is true
- ! *bool* — true if *bool* is false

Short-circuit Boolean Operators

Operators evaluate left-to-right. Operands are only evaluated if necessary, stopping once overall truth value can be determined. Operands are converted to scalars using the `all` function.

- *x* && *y* — true if both *x* and *y* are true
- *x* || *y* — true if at least one of *x* or *y* is true

Operator Precedence

Table of Octave operators, in order of increasing precedence.

- ; , — statement separators
- = — assignment, groups left to right
- || && — logical "or" and "and"
- | & — element-wise "or" and "and"
- < <= == >= > != — relational operators
- : — colon
- + - — addition and subtraction
- * / \ .* ./ .\ — multiplication and division
- ' .' — transpose
- + - ++ -- ! — unary minus, increment, logical "not"
- ^ .^ — exponentiation

Paths and Packages

`path`	display the current Octave cunction path.
`pathdef`	display the default path.
`addpath`(*dir*)	add a directory to the path.
`EXEC_PATH`	manipulate the Octave executable path.
`pkg list`	display installed packages.
`pkg load` *pack*	Load an installed package.

Cells and Structures

var.*field* = ...	set a field of a structure.
var{*idx*} = ...	set an element of a cell array.
`cellfun`(*f*, *c*)	apply a function to elements of cell array.
`fieldnames`(*s*)	returns the fields of a structure.

Statements

`for` *identifier* = *expr* *stmt-list* `endfor`

Execute *stmt-list* once for each column of *expr*. The variable *identifier* is set to the value of the current column during each iteration.

`while` (*condition*) *stmt-list* `endwhile`

Execute *stmt-list* while *condition* is true.

`break`	exit innermost loop
`continue`	go to beginning of innermost loop
`return`	return to calling function

`if` (*condition*) *if-body* [`else` *else-body*] `endif`

Execute *if-body* if *condition* is true, otherwise execute *else-body*.

`if` (*condition*) *if-body* [`elseif` (*condition*) *elseif-body*] `endif`

Execute *if-body* if *condition* is true, otherwise execute the *elseif-body* corresponding to the first `elseif` condition that is true, otherwise execute *else-body*.
Any number of `elseif` clauses may appear in an `if` statement.

`unwind_protect` *body* `unwind_protect_cleanup` *cleanup* `end`

Execute *body*. Execute *cleanup* no matter how control exits *body*.

`try` *body* `catch` *cleanup* `end`

Execute *body*. Execute *cleanup* if *body* fails.

Strings

`strcmp` (*s*, *t*)	compare strings
`strcat` (*s*, *t*, ...)	concatenate strings
`regexp` (*str*, *pat*)	strings matching regular expression
`regexprep` (*str*, *pat*, *rep*)	Match and replace sub-strings

Defining Functions

`function` [*ret-list*] *function-name* [(*arg-list*)]
function-body
`endfunction`

ret-list may be a single identifier or a comma-separated list of identifiers delimited by square-brackets.

arg-list is a comma-separated list of identifiers and may be empty.

Function Handles

@*func*	Define a function handle to *func*.
@(*var1*, ...) *expr*	Define an anonymous function handle.
`str2func` (*str*)	Create a function handle from a string.
`functions` (*handle*)	Return information about a function handle.
`func2str` (*handle*)	Return a string representation of a function handle.
handle (*arg1*, ...)	Evaluate a function handle.
`feval` (*func*, *arg1*, ...)	Evaluate a function handle or string, passing remaining args to *func*

Anonymous function handles take a copy of the variables in the current workspace.

Miscellaneous Functions

`eval` (*str*)	evaluate *str* as a command
`error` (*message*)	print message and return to top level
`warning` (*message*)	print a warning message
`clear` *pattern*	clear variables matching pattern
`exist` (*str*)	check existence of variable or function
`who, whos`	list current variables
`whos` *var*	details of the varibale *var*

Basic Matrix Manipulations

`rows` (*a*)	return number of rows of *a*
`columns` (*a*)	return number of columns of *a*
`all` (*a*)	check if all elements of *a* nonzero
`any` (*a*)	check if any elements of *a* nonzero
`find` (*a*)	return indices of nonzero elements
`sort` (*a*)	order elements in each column of *a*
`sum` (*a*)	sum elements in columns of *a*
`prod` (*a*)	product of elements in columns of *a*
`min` (*args*)	find minimum values
`max` (*args*)	find maximum values
`rem` (*x*, *y*)	find remainder of *x*/*y*
`reshape` (*a*, *m*, *n*)	reformat *a* to be *m* by *n*
`diag` (*v*, *k*)	create diagonal matrices
`linspace` (*b*, *l*, *n*)	create vector of linearly-spaced elements
`logspace` (*b*, *l*, *n*)	create vector of log-spaced elements
`eye` (*n*, *m*)	create *n* by *m* identity matrix
`ones` (*n*, *m*)	create *n* by *m* matrix of ones
`zeros` (*n*, *m*)	create *n* by *m* matrix of zeros
`rand` (*n*, *m*)	create *n* by *m* matrix of random values

Linear Algebra

`chol` (*a*)	Cholesky factorization
`det` (*a*)	compute the determinant of a matrix
`eig` (*a*)	eigenvalues and eigenvectors
`expm` (*a*)	compute the exponential of a matrix
`hess` (*a*)	compute Hessenberg decomposition
`inverse` (*a*)	invert a square matrix
`norm` (*a*, *p*)	compute the *p*-norm of a matrix
`pinv` (*a*)	compute pseudoinverse of *a*
`qr` (*a*)	compute the QR factorization of a matrix
`rank` (*a*)	matrix rank
`sprank` (*a*)	structrual matrix rank
`schur` (*a*)	Schur decomposition of a matrix
`svd` (*a*)	singular value decomposition
`syl` (*a*, *b*, *c*)	solve the Sylvester equation

Equations, ODEs, DAEs, Quadrature

`*fsolve`	solve nonlinear algebraic equations
`*lsode`	integrate nonlinear ODEs
`*dassl`	integrate nonlinear DAEs
`*quad`	integrate nonlinear functions
`perror` (*nm*, *code*)	for functions that return numeric codes, print error message for named function and given error code

* See the on-line or printed manual for the complete list of arguments for these functions.

Signal Processing

`fft` (*a*)	Fast Fourier Transform using FFTW
`ifft` (*a*)	inverse FFT using FFTW
`freqz` (*args*)	FIR filter frequency response
`filter` (*a*, *b*, *x*)	filter by transfer function
`conv` (*a*, *b*)	convolve two vectors
`hamming` (*n*)	return Hamming window coefficents
`hanning` (*n*)	return Hanning window coefficents

Image Processing

`colormap` (*map*)	set the current colormap
`gray2ind` (*i*, *n*)	convert gray scale to Octave image
`image` (*img*, *zoom*)	display an Octave image matrix
`imagesc` (*img*, *zoom*)	display scaled matrix as image
`imshow` (*img*, *map*)	display Octave image
`imshow` (*i*, *n*)	display gray scale image
`imshow` (*r*, *g*, *b*)	display RGB image
`ind2gray` (*img*, *map*)	convert Octave image to gray scale
`ind2rgb` (*img*, *map*)	convert indexed image to RGB
`loadimage` (*file*)	load an image file
`rgb2ind` (*r*, *g*, *b*)	convert RGB to Octave image
`saveimage` (*file*, *img*, *fmt*, *map*)	save a matrix to *file*

C-style Input and Output

`fopen` (*name*, *mode*)	open file *name*
`fclose` (*file*)	close *file*
`printf` (*fmt*, ...)	formatted output to `stdout`
`fprintf` (*file*, *fmt*, ...)	formatted output to *file*
`sprintf` (*fmt*, ...)	formatted output to string
`scanf` (*fmt*)	formatted input from `stdin`
`fscanf` (*file*, *fmt*)	formatted input from *file*
`sscanf` (*str*, *fmt*)	formatted input from *string*
`fgets` (*file*, *len*)	read *len* characters from *file*
`fflush` (*file*)	flush pending output to *file*
`ftell` (*file*)	return file pointer position
`frewind` (*file*)	move file pointer to beginning
`freport`	print a info for open files
`fread` (*file*, *size*, *prec*)	read binary data files
`fwrite` (*file*, *size*, *prec*)	write binary data files
`feof` (*file*)	determine if pointer is at EOF

A file may be referenced either by name or by the number returned from `fopen`. Three files are preconnected when Octave starts: `stdin`, `stdout`, and `stderr`.

Other Input and Output functions

`save` *file var* ...	save variables in *file*
`load` *file*	load variables from *file*
`disp` (*var*)	display value of *var* to screen

Polynomials

`compan` (*p*)	companion matrix
`conv` (*a*, *b*)	convolution
`deconv` (*a*, *b*)	deconvolve two vectors
`poly` (*a*)	create polynomial from a matrix
`polyderiv` (*p*)	derivative of polynomial
`polyreduce` (*p*)	integral of polynomial
`polyval` (*p*, *x*)	value of polynomial at x
`polyvalm` (*p*, *x*)	value of polynomial at x
`roots` (*p*)	polynomial roots
`residue` (*a*, *b*)	partial fraction expansion of ratio a/b

Statistics

`corrcoef` (*x*, *y*)	correlation coefficient
`cov` (*x*, *y*)	covariance
`mean` (*a*)	mean value
`median` (*a*)	median value
`std` (*a*)	standard deviation
`var` (*a*)	variance

Plotting Functions

`plot` (*args*)	2D plot with linear axes
`plot3` (*args*)	3D plot with linear axes
`line` (*args*)	2D or 3D line
`patch` (*args*)	2D patch
`semilogx` (*args*)	2D plot with logarithmic x-axis
`semilogy` (*args*)	2D plot with logarithmic y-axis
`loglog` (*args*)	2D plot with logarithmic axes
`bar` (*args*)	plot bar charts
`stairs` (*x*, *y*)	plot stairsteps
`stem` (*x*, `it y`)	plot a stem graph
`hist` (*y*, *x*)	plot histograms
`contour` (*x*, *y*, *z*)	contour plot
`title` (*string*)	set plot title
`axis` (*limits*)	set axis ranges
`xlabel` (*string*)	set x-axis label
`ylabel` (*string*)	set y-axis label
`zlabel` (*string*)	set z-axis label
`text` (*x*, *y*, *str*)	add text to a plot
`legend` (*string*)	set label in plot key
`grid` [on|off]	set grid state
`hold` [on|off]	set hold state
`ishold`	return 1 if hold is on, 0 otherwise
`mesh` (*x*, *y*, *z*)	plot 3D surface
`meshgrid` (*x*, *y*)	create mesh coordinate matrices

Edition 2.0 for Octave Version 3.0.0. Copyright 1996, 2007, John W. Eaton (jwe@octave.org). The author assumes no responsibility for any errors on this card.

This card may be freely distributed under the terms of the GNU General Public License.

TEX Macros for this card by Roland Pesch (pesch@cygnus.com), originally for the GDB reference card

Octave itself is free software; you are welcome to distribute copies of it under the terms of the GNU General Public License. There is absolutely no warranty for Octave.
